# Reconfiguration of life and work routines of senior professors during the COVID-19 pandemic

**DOI:** 10.1590/0034-7167-2024-0337

**Published:** 2025-08-08

**Authors:** Bianca dos Anjos Cavalini Rocha, Luiz Fernando Rangel Tura, Márcia de Assunção Ferreira

**Affiliations:** I Universidade Federal do Rio de Janeiro. Rio de Janeiro, Rio de Janeiro, Brazil

**Keywords:** Faculty, Aged, Pandemics, COVID-19, Nursing, Docentes, Anciano, Pandemias, COVID-19, Enfermería

## Abstract

**Objectives:**

to analyze the challenges and interferences experienced in reconfiguration of daily life and work of senior professors working remotely during the COVID-19 pandemic.

**Methods:**

qualitative research, with 15 professors from different levels of education. Snowball sampling and individual interviews were carried out, with structured and semi-structured questions. Analysis was simple statistical, percentage and lexicographic using Alceste software.

**Results:**

age ranged from 60 to 78 years, with a majority of women and difficulties in starting remote classes. There were more hours of work with overload, physical health problems due to a sedentary lifestyle and mental health problems.

**Final Considerations:**

learning to use technology was understood as reinventing oneself as a professor. Collaborative work was a strategy to overcome challenges, and there was little psychological support from institutions. There is a clear need for investment in updating teaching staff and greater assistance to older teachers in their difficulties to overcome limitations.

## RESUMEN

**Objetivos:**

analizar los desafíos e interferencias vividas en la reconfiguración de la vida cotidiana y el trabajo de los docentes adultos mayores que trabajan en la enseñanza remota durante la pandemia de COVID-19.

**Métodos:**

investigación cualitativa, con 15 docentes de diferentes niveles docentes. Se realizó muestreo en bola de nieve y entrevistas individuales, con preguntas estructuradas y semiestructuradas. El análisis fue estadístico simple, porcentual y lexicográfico mediante el software Alceste.

**Resultados:**

las edades oscilaron entre 60 y 78 años, siendo la mayoría mujeres y con dificultades para iniciar clases a distancia. Hubo más horas de trabajo excesivo, problemas de salud física debido al sedentarismo y problemas de salud mental.

**Consideraciones Finales:**

aprender a manejar las tecnologías se entendió como reinventarse como docente. El trabajo colaborativo fue una estrategia para superar desafíos y hubo poco apoyo psicológico por parte de las instituciones. Es evidente la necesidad de inversiones en la actualización del personal docente y de una mayor asistencia a los docentes de edad avanzada en sus dificultades para superar las limitaciones.

## INTRODUCTION

There are older adults who need support to carry out daily activities, with a high degree of dependence and generating higher health costs^(1)^. However, although older adults are part of a population that needs a support network, not because they are dependent, but because they are in an age group with specific demands, the changes in the contemporary world have created new views on older adults. Many work or are available to work, maintain an active social life, have relationships, consume products and services, travel and maintain independent social activities. In contemporary society, older adults are active and generate income for the country, in addition to being healthy to have a long life, reducing health costs in general^(2)^.

In Brazil, healthy life expectancy at age 65 was 11.4 years in 1990 and rose to 13.2 in 2019 and this criterion serves as a guide to assess healthy longevity^(3)^. The beginning of the 21^st^ century brought profound changes to scientific and technological knowledge that were incorporated by the productive market, triggering complex cultural inequalities, facilitating access to knowledge but widening the space between people^(4)^. The development of new technologies influences society’s way of life, especially in communication and social interaction. It is challenging for older adults to adapt to new demands, but despite the difficulties, their use brings benefits to this age group.

With the COVID-19 pandemic, daily life has undergone major changes, with reconfigurations in the world of work. Remote teaching was implemented to avoid paralyzing educational processes and school and professional training. Institutions and their workers have reorganized themselves, with new demands for professors to adapt to the new format and improvise their home space, sharing their professional and family lives^(5)^.

For most senior professors, remote teaching was something new and difficult to practice, as they were not formally prepared for this modality, nor did they have the skills to deal with the resources and appropriate teaching methods. The strategies were designed on an emergency basis, based on resources available, but they required adaptations that in many cases caused distress and anguish due to the lack of preparation for this teaching modality. It was necessary to rethink the school and its participants, including the family, requiring everyone’s cooperation in the process^(6)^.

The pandemic has highlighted the lack of research on senior professors’ experiences in remote teaching, but this does not mean that senior professors do not experience problems in this modality^(7)^. In the Philippines, senior professors faced challenges and health problems, needed support and had great resilience to meet the demands^(8)^. However, research conducted in the United States of America showed that senior professors dedicated themselves to remote teaching to overcome technical and digital obstacles and made an effort to learn the new things that were presented, despite concerns about potential burnout^(9)^.

As a novelty, remote work made possible by electronic/digital technologies is likely to generate social representations (knowledge and practices)^(10)^, especially for senior professors, who needed to reconfigure their ways of communicating, develop new knowledge about technologies, learn about them and learn to deal with them in their daily lives. Accessing their knowledge and learning about the practices of reconfiguring senior professors’ daily lives in the face of remote teaching implemented during the pandemic is relevant to generate knowledge that broadens the understanding of social inclusion of older adults in the contemporary world, especially in teaching work when social distancing is required with restrictions on meetings and physical contact.

## OBJECTIVES

To analyze the challenges and interferences experienced in reconfiguration of daily life and work of senior professors working remotely during the COVID-19 pandemic.

## METHODS

The project was approved on March 28, 2022 by the Research Ethics Committee of the *Escola de Enfermagem Anna Nery* and *Instituto de Saúde São Francisco de Assis* at the *Universidade Federal do Rio de Janeiro*. The main researcher introduced herself to potential participants, informed them about the research and, upon acceptance, all signed the Informed Consent Form.

### Theoretical-methodological framework

The applied framework is that of the Theory of Social Representations, considering the concepts of objectification and anchoring in the formation of social representations^(10,11)^.

### Study design

This is a qualitative and descriptive study, applying the COnsolidated criteria for REporting Qualitative research (COREQ) guidelines in the development of its stages.

### Methodological procedures

The snowball sampling method was applied, since no specific settings were initially chosen to gather the study’s target population, and the initial probabilistic sampling was difficult to practice^(12)^. Two seeds were contacted, both from the city of Rio de Janeiro, one from elementary school, indicated by the main researcher’s social and personal network, and the other from higher education, indicated by one of the researchers on the team. From these, new participants were indicated. Data collection ended with 15 participants, following the data saturation criterion^(13)^.

### Data source

Inclusion criteria were professors aged 60 or over who had worked in remote teaching during the COVID-19 pandemic, using electronic equipment and the internet in online classrooms. Exclusion criteria were professors who had worked in distance or hybrid teaching modalities before 2020, and who had been away from work due to illness that prevented them from participating in online interviews. There were no exclusions or withdrawals of any of the participants.

### Data collection and organization

The main researcher was responsible for data collection, from May 2022 to July 2023. Individual interviews were conducted remotely and video and voice recordings were performed, with an average duration of 30 minutes, with a script with structured questions (to characterize the psychosociodemographic profile regarding age, level of education and training, marital status, who the person lives with, whether they had difficulties dealing with information and communication technologies (ICTs) platforms and in remote teaching, daily working hours) and a script with semi-structured questions (about ways of dealing with ICTs, facilities and difficulties, solutions, meeting needs, relationship with students, routines and daily work). The following questions were explored: who knows, where they know from, what they know, how they know and the effect^(10)^.

### Data analysis

The psychosociodemographic profile data analysis was performed using absolute and relative frequencies. The transcribed interviews formed the *corpus* of qualitative data with 15 Initial Context Units (ICU). This single text was processed using Alceste software, which applies statistical tests to track words to perform contextual lexical analysis of a set of text segments. These tests look for the frequency and co-occurrence of words in processed speeches, separating text segments that make sense in the explanations of ideas conveyed by participants about the object/phenomenon investigated. In this way, different collective points of view are identified that make it possible to capture social representations^(14)^.

By statistically associating words in text segments, the software generates dendrograms called Descending Hierarchical Classification (DHC), which hierarchizes the most significant words in lexical classes based on a Phi value and forms lists with the reduced forms (roots) and corresponding full forms. Words are understood by reading the text segments to which they belong, called Elementary Context Units (ECU). The objective was achieved by analyzing two lexical classes. Class 1 deals with the challenges of adapting to a new life routine in the face of the pandemic, and class 2 addresses the interferences experienced by senior professors during the pandemic, which are the categories of analysis.

## RESULTS

The research was mostly conducted by women (13-87%) and only two (13%) were men. Their ages ranged from 60 to 78 years old. Three (20%) are high school professors; one (7%) is an elementary school professor; and 11 (73%) are in higher education. Nine (60%) have *stricto sensu* graduate degrees; nine (60%) work exclusively in public institutions; 13 (87%) work more than eight hours a day; and 11 (73%) have higher education. Concerning marital status, nine (60%) are married; four (26%) are divorced; one (7%) is a widow; and one (7%) is single. Regarding the nuclear family, nine (60%) live with their spouse; one (7%) lives with their children; three (20%) live alone; and two (13%) live with other people. Most participants reported difficulties in starting classes remotely (11-73%).

Lexical classes 1 and 2 deal with how the COVID-19 pandemic impacted people’s routines, especially senior professors. Workers had to readapt and reorganize their personal lives, associating them with their professional lives, with working from home. These classes portray the professors’ routine and how they associated taking care of their health with the dynamics adopted in developing remote classes. To understand them, the list of ECUs belonging to it was analyzed to understand the contexts in which the lexicons were inserted, considering the relationship between words expressed through DHC.

The challenges of adapting to a new routine were most evident in class 1, with 18% (85) of the total ECUs in the *corpus* analyzed and 119 reduced forms of full words. The analysis of lexicons associated with class 1 showed that ECUs deal with professors’ routine during the pandemic to readjust their new work model. As a result, some ECUs present words that express comfort, agility and pleasure of being at home and working at the same time, while others refer to health problems due to the break in routine and spending too much time in the same position, for instance. The lexicon most associated with this class is “hour”, with Phi 0.37, as can be seen in DHC shown in Chart 1.

**Chart 1:** Representative words of class 1 according to the Descending Hierarchical Classification dendrogram generated by Alceste

Class 1 - 85 Elementary Context Units	
**Full form and frequency**	**Phi value**
hour (15) hours (23)	0.37
time (43)	0.27
leave (12)	0.27
home (32)	0.26
season (7)	0.26
schedule (1) schedule (7) schedules (2)	0.25
lunch (1) lunch (6)	0.24
works (2) work (1) working (5) to work (6) worked (3)	0.24
physics (13) physics (1)	0.23
commute (9)	0.23
ends (3) ended (3) ended (2) ended (3)	0.23
prepare (8) preparation (2)	0.22
way (5)	0.22
night (5)	0.22
eat (3) eat (5)	0.21
street (6)	0.21
search (4) searched (1) search (2)	0.21
wake (2) wake (3)	0.19
start (2) started (1) started (1) started (4) started (2)	0.19
day (15) days (1)	0.19
leave (2) skirt (3)	0.19
walk (3) walk (1) walk (2) walked (2)	0.19
activity (13) activities (5)	0.19
go (9)	0.17
arrive (1) arrive (3) arrived (4) arrived (1)	0.17
life (6) lives (1)	0.17
run (2) ran (1)	0.17

*Source: Alceste detailed report, 2023.*

The pandemic and the shift from the institutional classroom to the residential environment implied a significant readjustment of professors’ routines during the pandemic, with many hours of work and screen time.

*We are at home, we don’t waste time, we go and have coffee and get up every hour. Normally, meetings started at nine. I spent a lot of time on the computer, it got bad. Practically the whole day was in meetings, it was super busy.* (ICU 13)

*Literally working. Sometimes it was seven in the morning and I was sitting in front of the computer working, and at lunchtime, I often ate, I would close the camera and we would continue working.* (ICU 7)

The readjustment of routines has generated work overload and reorganized daily self-care habits, especially with regard to diet and physical activities. In this regard, physical and mental health during and after the pandemic are aspects to be considered in relation to professors’ occupational health, especially because they are older adults.

[In-person classes] *All day long, you go up and down the stairs; you go to one place; you go to another; you eat at the times you have to eat. In remote learning, you go to the kitchen every hour, you eat something out of time, you don’t do any physical activity properly, you don’t have a routine. I did absolutely no physical activity. I would get up from my chair to eat and drink water, it was horrible. As soon as it was allowed, I went back to doing activities.* (ICU 14)

*I stopped walking. I had back pain, my calf was injured, shortened, I almost couldn’t walk because I was sitting, I couldn’t walk.* (ICU 7)

*Sometimes, ten o’clock at night and I was working, scheduling student orientation meetings at eight, nine o’clock at night, because they had other activities. This caused physical exhaustion and also a bit of mental exhaustion*. (ICU 10)

Among the challenges with technology, senior professors had to deal with new plans in light of the reorganization of schedules to cope with the new work reality they were experiencing.

*Planning my schedule is not the same as in the classroom. I get excited, I start doing something and forget to eat. I used to forget everything. I sit there and go, because if I stop, it seems like I’ll lose focus and motivation, because there’s still a lot to learn. It’s making the work easier. It’s a good thing we were in the middle of the pandemic, I had the whole day at home, because sometimes I’ll prepare a class, it would take me a whole day, and I’d spend part of the night researching, because I only know how to work with books.* (ICU 6)

The results show a dialectic in the new way of teaching, with positive and negative aspects. On the one hand, working remotely allows professors to save time on commuting, but on the other, confinement was not a desired state.

*Sometimes I would lie in bed and wonder when we would go back to that world of traffic jams and the two-hour commute to work. Will we ever get used to it again? But at the same time, we didn’t want to stay that way.* (ICU 13)

Saving time has two sides, as what is left over from the journey that did not take place results in more working hours.

*Because you spent a lot more time working; you had to get ready to get to and from work and get home. I don’t spend time getting ready, putting on makeup, looking for clothes, shoes, going out and catching the subway, so what do we use the time I used for that? Literally working.* (ICU 7)

Professors also indicated that, for students, remote teaching brought some advantages, such as the possibility of studying at times and places that are not the norms for in-person teaching, in addition to enabling financial savings on travel, increasing the possibility that more students could be included in educational institutions.

*The high cost of getting to and from college. Remote learning can be good because it can include many more students in college. The bad thing is not having a proper routine, eating too much, gaining too much weight, changing your habits, because, on a daily basis, you have to rush to wake up, get ready, have breakfast, sometimes even get in the car and go to work.* (ICU 14)

However, they believe that students need to readapt to in-person teaching after the pandemic, with the discipline that the physical space of the classroom requires.

*They* [students] *are relearning how to live in society; they can’t spend so many hours sitting in the same place, because at home, they were on the sofa, in bed; they sat any which way, now they have to spend five hours sitting, everything is more difficult.* (ICU 6)

The interference experienced by professors during the pandemic was most evident in class 2, which has 42% (192) of the total ECUs that constitute the *corpus* of analysis, making it the densest class. It consists of 135 reduced forms of full words found. The analysis of lexicons associated with class 2 highlighted the mechanisms used by both professors and students to communicate and exchange information in light of the new classroom model. Words such as microphone, online calls, open windows, and slow internet are seen as factors that directly interfered with daily life. The lexicon most associated with this class is class, as can be seen in Chart 2.

**Chart 2:** Representative words of class 2 according to the dendrogram of the Descending Hierarchical Classification generated by Alceste

Class 1 - 192 Elementary Context Units	
**Full form and frequency**	**Phi value**
class (94) classes (47)	0.32
question (5) asking (1) asking (2) asked (1) questions (5)	0.24
students (59)	0.22
platform (16) platforms (6)	0.23
*online (37)*	0.21
send (5) send (2) sent (1) sent (2) sent (1) send (2)	0.18
used (18) used (1)	0.16
course (14) courses (2)	0.16
start (2) started (2) start (13) started (2)	0.15
doing (17)	0.15
support (7)	0.14
interact (3) interacted (1) interacted (2) interact (1)	0.14
speak (3) speak (2) speaking (7) speak (6) spoke (10) spoke (3) spoke (4) speak (2) spoke (2)	0.13
student (1) students (1) student (35)	0.13
enter (2) entrance (2) entered (2) enter (7) entered (4) entered (2) enter (3)	0.13
bed (1) camera (17)	0.12
river (6)	0.13
room (25) rooms (4)	0.12
jitsi (7)	0.13
virtual (2) virtual (13)	0.12
confusion (4) confusing (3)	0.13
*notebook (10)*	0.13
sharing (1) shared (1) sharing (1) share (6)	0.13
learning (9)	0.13

*Source: Alceste detailed report, 2023.*

Some of the challenges reported by professors in teaching remote classes concern students’ private lives, which came to light in the remote classroom, as their inability to use microphones brought into the classroom part of what was happening in their homes. Others report difficulties and inabilities of everyone, students and professors, with the platform’s tools, requiring learning to better handle and take advantage of the classes.

*They forgot to turn the microphone on; children would cry, dogs would bark, and that was part of the incident, the microphone on. Other than that, the students performed very well because they paid attention. If the microphone was on, I would ask them to turn it off; they would obey, pay attention, and interact in the classroom.* (ICU 11)

*In the beginning, it was all very confusing. The students couldn’t find the right classrooms, they couldn’t connect, they were late for classes, it wasn’t a very good experience. They had online courses and sent us materials to help us, they gave us support.* (ICU 14)

*The notices were there, but I wasn’t the one who put them up, the course coordinator was, because I only knew how to go in and look. On the Jitsi platform, I learned quickly, how to go in and share the screen, put it on full screen. At first, I had difficulty.* (ICU 8)

*Sometimes the image is bad. It was a shock for me, but now I look back on it with a positive outlook. I reinvented myself. And with online classes, as I said, I need to be vigilant, because if a student is doing the exercise wrong, I need to call their attention to it.* (ICU 3)

*In fact, in the classes, there was a person from the school’s IT department who was there for the first few classes. If we had any difficulty using the equipment, they would give us support until we learned how to do it.* (ICU 9)

In the face of adversity, reinventing oneself was the strategy applied to fulfill work activities during the pandemic. This “reinvention” was done with the help of colleagues and with learning at the same time as work.

*Those who were more laid back would say, “You know where I come in, how I’m going to share, how I connect this?”. It was during development and we finally got it, but the virtual learning environment is good, but it’s a bit complicated. I didn’t teach in this environment.* (ICU 13)

*There was little psychological support from the institution. Sometimes it’s needed. There’s always someone at the school to help, to provide support. We have a psychologist here, so we run to him, send the student who is having problems, to talk to the student.* (ICU 6)

*We had groups on WhatsApp. We helped each other, we tried to do the classes in pairs, and one would interact with the class in the chat and the other would teach the class.* (ICU 14)

Concerns about students’ ability to take advantage of classes and tests and the realization that being logged in and online did not mean being present in class.

*Some students seemed to be online, and there were classes where the coordinator would not accept that only their faces were present. We have to be online, showing the movements of each one of* you [referring to graduate students]; *she demanded that it be that way. In remote teaching, she noticed that the student was not present, only their face was there.* (ICU 9)

## DISCUSSION

To control the COVID-19 pandemic, social distancing measures were adopted, with restricted movement of people and implementation of remote work^(15)^. What we saw was a readaptation of the work routine both for professionals who remained in person and for those who started working remotely^(16)^.

Those who continued in person were afraid of contagion, but they were also afraid of unemployment and tried to continue with their routine, considered “normal”. Those who worked remotely had difficulty adapting, with changes in the organization and routine of their home, understood as a place of restoration and rest, became their new workspace, in addition to the difficulties in balancing it with domestic demands^(16)^. Administrative and school services were those that most adopted this type of work^(15)^. The closure of activities had a strong impact on educational institutions. This required all levels of education to work remotely on an emergency basis^(17)^.

Since the beginning of the pandemic, people have had to readjust their lives and experience sudden and significant changes in their daily lives. The time spent preparing for and commuting to in-person workspaces has been invested in the work itself, which has changed the routine of senior professors. Regarding health behaviors, it is observed that taking care of one’s lifestyle is a recommendation to maintain and promote older adults’ health, with physical exercise, diet, rest and leisure being important pillars to be protected^(18)^. Precisely such care was compromised with the implementation of remote work, being one of the negative aspects of this phase, in light of representations of senior professors.

A survey conducted on sedentary behavior during the COVID-19 pandemic, with adults and older adults from Campo Grande, in Mato Grosso do Sul, had the participation of 1,907 people and showed excessive use of screens by 86.7% of participants in general, with older adults showing a lower frequency of screen time, but a higher proportion of time spent sitting^(19)^. This research was not conducted with professors in remote teaching, and its citation is not intended to establish comparisons with research results that is the subject of this article. However, it points to the tendency of older adults to be sedentary during the pandemic and to spend a lot of time sitting, a behavior typical of those who work for many hours on computers, which indicates a low load of physical activities during this period of confinement at home.

A survey of 1,115 Dutch professors from all levels of education, the majority of whom were female (76.7%) and aged between 19 and 69 years (average age 43 years), indicated that their personal lives were compromised by remote work, highlighting the need for a balance between the world of work and the private world in terms of working hours, better facilities for working from home and reconciling other responsibilities and self-care^(20)^. In Brazil, research with 17 professors at a public university showed that changes in work routines made it difficult to limit working and resting hours^(21)^.

The impact on mental health and the need for psychological support emerged as one of the concerns of senior professors. Epidemics have a major impact on mental health and in the case of COVID-19, the lack of breaks and rest was identified as one of the factors for this impairment^(22)^. A balanced routine between taking care of oneself, work and family, maintaining physical activities, as long as preventive care against infection is taken, are recommended measures to maintain good mental health^(22)^. Senior professors in remote teaching highlighted that daily routine, overwork and sedentary lifestyle compromised their daily lives. Research carried out on the social representations of confinement with professionals and students captured through social networks showed that the effects of confinement were felt in everyone’s mental health, but especially in the group of students^(23)^, which indicates that this is a burning topic for studies at the intersection of the fields of education and health.

In addition to daily adaptations, structural reorganizations related to the reconfiguration of teaching work required learning new skills to deal with the technologies that remote work requires^(24)^. The re-adaptation and reinvention of teaching methods to teach classes implied changes in daily life at home, as virtual classes altered personal routines. Many reported the need to learn how to use and access technological platforms and encountered connection problems, students without the possibility of accessing them, difficulties communicating due to external noise, among others.

In line with the problems experienced by senior professors, Russian professors who worked in remote teaching also indicated that the lack of preparation to deal with digital technologies, resources and time to prepare were the main challenges to face in the emergency remote teaching required in the recent pandemic^(20)^. Professors and students had difficulties and this led to interference of various kinds, mainly due to open microphones and delays in the start of activities. Teaching work in itself generates many demands^(24)^ and when professors find themselves in a situation where they need to relearn their craft in the face of technologies that they have not mastered after years of work, and even in an adverse context such as a pandemic, emotions surface. First comes fear, then acceptance and reinvention.

Social representations are almost tangible, they circulate in speech, gestures, encounters and permeate the everyday universe, corresponding to the symbolic substance of their elaboration and the practice that produces such substance^(11)^. This is practical knowledge that is organized and constructed in response to new developments in everyday life, mobilizing emotions and influencing behavior. This construction occurs through anchoring processes, which assimilate the new development into the existing system of thought, and through objectification, which, upon accepting this new development, integrates it into everyday actions^(10)^.

Professors were faced with the novelty of a new way of working, in new spaces and with tools they were already familiar with - computers - but not as a classroom. Accustomed to physical classrooms, they had to move to the virtual environment and deal with this new situation, which presented itself as a novelty that demanded new ways of acting and how to behave in this new space, with the transformation of their knowledge about how to teach classes in a non-physical space for students who are not always visible to them.

Moving from the physical classroom to the virtual one required special attention, with concerns that professors did not have before: being used to students participating only through voice, since many did not turn on their cameras, which required new learning about presence based on the sense of hearing rather than sight; checking for connectivity problems; and adapting class proposals to the resources available on the platform used by the institution.

Teaching work is not limited to the classroom and it is worth highlighting that in addition to the new demands, those already existing in the scope of teaching work have been added, especially when working in higher education, which include leading research groups, supervising academic work, coordinating extension projects, among others^(25)^.

The issue of remote learning enabling greater student inclusion was highlighted in the context of saving on travel costs. However, school closures have entailed high social and economic costs for the population in different spheres^(26)^. The impact has been particularly severe for the most vulnerable and marginalized and their families, with learning disrupted and opportunities for growth and development lost. The disadvantages are disproportionately felt by less privileged students, who tend to have fewer educational opportunities beyond school.

In Brazil, most public schools were closed for almost the entire school year in 2020 and only reopened in 2021, unlike private schools, which reopened in September and October 2020, which highlights the inequalities in access for students^(27)^. Students in public schools, from elementary to higher education, felt the impact of the sudden change in classes much more, due to the lack of technological equipment and internet access in their homes, in addition to difficulties in using applications. A worrying aspect is the greater impact of the pandemic on students considered vulnerable and marginalized, who may fall further behind^(28)^. One of the topics that the sociology of education discusses is the relationship between the socioeconomic level of students and academic success, since social background has an important meaning in student performance.

The results presented are consistent with those of a survey carried out with professors from a higher education institution in southern Brazil which, despite not being aimed at senior professors, indicated challenges similar to those faced by older adults regarding the teaching-learning process, the demand for learning new digital technologies and the impacts on professors’ mental health^(29)^.

Overcoming fear and insecurity to learn how to deal with technology permeated the experiences of professors in remote teaching, and interference in private life, work and leisure marked the experiences of southern professors as well as of the senior professors participating in this research. Difficulties with the use of equipment and digital platforms were felt by different professors, fear, anguish and uncertainty about what teaching work would be like remotely accompanied them on this journey of familiarization and readaptation of work^(30)^. In relation to the phenomenon of fear, it is worth noting that fear of the unknown is a specific type that takes hold of people in times of crisis^(31)^. As an affective state, fear structures the response so that a person adapts to situations, which, being rational, allows reactions to situations that present themselves as new^(31)^.

The strategies used to develop quality work that would fulfill the purpose of teaching and learning also needed to be reformulated, since the teaching space has changed. Verifying the effective presence of students in the virtual environment and strengthening solidarity among professors were highlighted. Doubts and uncertainties regarding the context and the new teaching modality mobilized feelings/emotions in the production of meanings about remote work, activating prior knowledge, norms, values, ideologies and belief systems that are shared socially. We can see the role that emotions play in cognitive work in the mobilization of social knowledge to reinterpret the world and in transactions (agreements, practices) with the environment^(31)^. The quality of remote teaching is reflected in professors’ perceptions^(21,32)^. Professors and pedagogical coordinators of a master’s degree course at a university in the city of São Paulo expressed concerns about students’ learning due to different profiles of age inequality, curriculum, teaching strategies and internet access^(32)^.

The sense of empathy and solidarity towards other professors was felt in this confrontation. School management and relationships with colleagues emerged from the needs of Dutch professors working remotely during the pandemic, with more guidance from institutions regarding the work process itself in this new modality and concern for colleagues who need help and support, in a more collaborative work^(20)^.

The difficulties with technologies and their representation as a challenge permeate the set of results of this and other research studies carried out during the pandemic on the subject. The pandemic brought to light the tense relationship between the work of senior professors and remote teaching technologies. To support this statement, a study carried out in the Philippines on news broadcast in videos involving senior professors and emergency online learning implemented during the COVID-19 pandemic showed that professors made an effort to adapt to this type of teaching, striving to learn how to use the tools and meet expectations. The authors indicate that, in the Philippines, senior professors represent one of the challenges to be faced by school administration and the government. These professors need more attention in order to achieve and improve technical skills and confidence to work in remote teaching^(8)^.

The complexity of one of the platforms used by the institution was translated as a complicated environment that culminated in the option of not using it. This situation was also highlighted in the research carried out in the Philippines, as one of the recommendations was the production of easy-to-use platforms, to help senior professors in this teaching modality^(8)^.

Research into theses and dissertations produced between 2017-2021 on continuing education for professors and digital technologies analyzed 44 works, half of which were produced in the southeast region, focusing on pedagogical practices in the classroom, which shows basic education as the centrality of studies^(33)^. Therefore, continuing education is a good strategy to prepare professors to use digital resources, not only for remote teaching, which had an emergency nature during the pandemic, but because the digital field in education is a path that only tends to grow.

### Study limitations

This research was conducted with professors from a municipality in the southeast region. Brazil is a continental country, and a broader debate with the experience of senior professors from other regions would allow us to consider diverse sociocultural realities and more comprehensive proposals for the area.

### Contributions to nursing, health or public policy

Population aging is a contemporary reality, as are technological advances, despite information and communication resources. Education is an area of important application of technologies to diversify teaching-learning strategies, whether for remote or distance learning, and to disseminate information and popularize science. Including senior professors in this debate and preparing them for these practices is timely and necessary, given the contingent of people aged 60 or over who are present in the job market, and this recognition is fundamental for the qualification and health of this population group.

## FINAL CONSIDERATIONS

The adjustment of routine implied more working hours, since the savings in commuting time resulted in work overload. The loss of routine resulted in increased food consumption and weight gain, sedentary lifestyle and compromised mental health. The challenges of the new classroom space were faced by learning the platform’s functionalities, translated as a process of “reinventing” oneself as a professor in the development of the work. There was little psychological support from the institutions. The inclusion of students, due to savings in commuting costs and collaborative work were positive aspects. There is a clear need for investment in updating the teaching staff and greater assistance to senior professors in their difficulties in overcoming limitations.

## Data Availability

Not applicable.
