# Supplementary material for: Reconfiguration of life and work routines of senior professors during the COVID-19 pandemic
Source: Rev Bras Enferm. 2025 Aug 8;78(Suppl 2):e20240337. doi: 10.1590/0034-7167-2024-0337 (PMC12334130; doi:10.1590/0034-7167-2024-0337)
Supplement: Translation

# Translation: Reconfiguração das rotinas de vida e de trabalho de docentes idosos na pandemia de COVID-19

**DOI:** 10.1590/0034-7167-2024-0337pt

**Authors:** Bianca dos Anjos Cavalini Rocha, Luiz Fernando Rangel Tura, Márcia de Assunção Ferreira

**Affiliations:** I Universidade Federal do Rio de Janeiro. Rio de Janeiro, Rio de Janeiro, Brasil

**Keywords:** Docentes, Idoso, Pandemias, COVID-19, Enfermagem

## Abstract

**Objetivos:**

analisar os desafios e interferências vivenciadas na reconfiguração do cotidiano de vida e de trabalho de docentes idosos atuantes no ensino remoto na pandemia de COVID-19.

**Métodos:**

pesquisa qualitativa, com 15 docentes de diferentes níveis de ensino. Realizaram-se amostragem de bola de neve e entrevista individual, com perguntas estruturadas e semiestruturadas. A análise foi estatística simples, percentual e lexicográfica pelo *software* Alceste.

**Resultados:**

a idade variou de 60 a 78 anos, sendo majoritariamente mulheres e com dificuldades para iniciar as aulas remotas. Houve mais horas de trabalho com sobrecarga, problemas de saúde física pelo sedentarismo e mentais.

**Considerações Finais:**

aprender a manusear as tecnologias foi entendido como se reinventar como docente. O trabalho colaborativo foi estratégia para vencer desafios, e houve pouco apoio psicológico das instituições. Evidencia-se a necessidade de investimentos em atualizações do corpo docente e maior assistência aos docentes idosos nas suas dificuldades para vencer as limitações.

## INTRODUÇÃO

Há pessoas idosas que necessitam de apoio para cumprirem atividades cotidianas, com alto grau de dependência e geradores de maiores custos à saúde^(1)^. No entanto, ainda que os idosos integrem uma população que necessita de rede de apoio, não por serem dependentes, mas por estarem em uma faixa etária com demandas específicas, as transformações do mundo contemporâneo imprimiram novas visões sobre a pessoa idosa. Muitas exercem atividades laborais ou têm disponibilidade para o trabalho, mantêm vida social ativa, relacionam-se, consomem produtos, serviços, viajam e mantêm atividades sociais independentes. Na sociedade contemporânea, pessoas idosas são ativas e geram renda para o país, além de serem saudáveis para obter vida longa, reduzindo gastos para a saúde de um modo geral^(2)^.

No Brasil, a expectativa de vida saudável aos 65 anos de idade era de 11,4 anos, em 1990, e subiu para 13,2, em 2019, e este critério serve de guia para avaliar a longevidade saudável^(3)^. O início do século XXI trouxe mudanças profundas para o conhecimento científico e tecnológico que foram incorporadas pelo mercado produtivo, com desencadeamento de desigualdades culturais complexas, facilitando o acesso ao conhecimento, mas alargando o espaço entre as pessoas^(4)^. O desenvolvimento de novas tecnologias influencia o modo de vida da sociedade, em especial na comunicação e interação social. Para a pessoa idosa, é desafiador adaptar-se às novas exigências, porém, apesar das dificuldades, seu uso traz benefícios para este grupo etário.

Com a pandemia de COVID-19, a vida cotidiana sofreu importantes alterações, com reconfigurações no mundo do trabalho. O ensino remoto foi implantado para não paralisar os processos educacionais e de formação escolar e profissional. As instituições e seus trabalhadores se reorganizaram, com novas exigências para que os docentes se adaptassem ao novo formato e improvisassem o espaço doméstico, compartilhando sua vida profissional e familiar^(5)^.

Para a maioria dos docentes idosos, o ensino remoto foi algo novo e difícil de praticar, por não estarem formalmente preparados para essa modalidade, pelas habilidades para lidar com os recursos e a didática apropriada. As estratégias foram pensadas, emergencialmente, com base nos recursos disponíveis, mas precisaram de adaptações que, em muitos casos, geraram sofrimento e angústia pela falta de preparo para esta modalidade de ensino. Foi preciso repensar a escola e seus partícipes, incluindo a família, requerendo a cooperação de todos no processo^(6)^.

A pandemia trouxe à tona a escassez de pesquisas sobre a experiência de docentes idosos no ensino por meio remoto, mas isso não significa que docentes idosos não vivenciem problemas nessa modalidade^(7)^. Nas Filipinas, professores idosos enfrentaram desafios e problemas de saúde, necessitaram de apoio, e tiveram muita resiliência para atender às demandas^(8)^. Não obstante, pesquisa realizada nos Estados Unidos da América mostrou que docentes idosos se dedicaram ao ensino remoto para vencer obstáculos técnicos e digitais, e se esforçaram no aprendenzidado das novidades que se apresentaram, apesar das preocupações com potenciais esgotamentos^(9)^.

Como uma novidade, o trabalho remoto viabilizado por tecnologias eletrônicas/digitais é passível de gerar representações sociais (saberes e práticas)^(10)^, especialmente para docentes idosos, que precisaram reconfigurar os modos de se comunicar, desenvolver novos saberes sobre as tecnologias, apreendê-las e aprender a lidar com elas em seus cotidianos. Acessar seus saberes e conhecer as práticas de reconfiguração do cotidiano de docentes idosos em face do ensino remoto implantado na pandemia é relevante para gerar conhecimentos que ampliem a compreensão sobre a inserção social da pessoa idosa no mundo contemporâneo, em especial no trabalho docente, quando se exige distanciamento social com restrição de encontros e contato físico.

## OBJETIVOS

Analisar os desafios e as interferências vivenciadas na reconfiguração do cotidiano de vida e de trabalho de docentes idosos atuantes no ensino remoto na pandemia de COVID-19.

## MÉTODOS

Projeto aprovado em 28 de março de 2022 pelo Comitê de Ética em Pesquisa da Escola de Enfermagem Anna Nery e do Instituto de Saúde São Francisco de Assis da Universidade Federal do Rio de Janeiro. A pesquisadora principal se apresentou aos potenciais participantes, informou sobre a pesquisa, e, mediante o aceite, todos assinaram o Termo de Consentimento Livre e Esclarecido.

### Referencial teórico-metodológico

O referencial aplicado é o da Teoria das Representações Sociais, considerando os conceitos de objetivação e ancoragem na formação de representações sociais^(10,11)^.

### Tipo de estudo

Estudo qualitativo, descritivo, com aplicação das diretrizes do *COnsolidated criteria for REporting Qualitative research* (COREQ) no desenvolvimento de suas etapas.

### Procedimentos metodológicos

Aplicou-se o método bola de neve, uma vez que não se elegeram, a princípio, cenários específicos para a captação da população-alvo do estudo, sendo a amostragem probabilística inicial difícil de ser praticada^(12)^. Duas sementes foram contatadas, ambas da cidade do Rio de Janeiro, sendo uma do ensino fundamental, indicada pela rede de relações sociopessoais da pesquisadora principal, e a outra do ensino superior, indicada por uma das pesquisadoras da equipe. A partir dessas, novos participantes foram indicados. Encerrou-se a coleta de dados com 15 participantes, seguindo-se o critério de saturação dos dados^(13)^.

### Fonte de dados

Os critérios de inclusão foram professores com 60 anos ou mais de atuação no ensino remoto no período da pandemia de COVID-19, com uso de equipamentos eletrônicos e internet em salas de aula *online*. Os critérios de exclusão foram atuação nas modalidades de ensino a distância ou híbrido antes de 2020, afastados do trabalho por motivos de adoecimento que os impedissem de participar de entrevistas *online*. Não houve exclusões e nem desistências de nenhum dos participantes.

### Coleta e organização dos dados

A pesquisadora principal responsabilizou-se pela coleta, de maio de 2022 a julho de 2023. Realizaram-se entrevistas individuais por meio remoto e gravação de vídeo e voz, com duração média de 30 minutos, com um roteiro com perguntas estruturadas (para a caracterização do perfil psicosociodemográfico sobre a idade, nível de formação e de ensino, estado conjugal, com quem habita, se teve dificuldades para lidar com as plataformas e tecnologias de informação e comunicação (TICs) no ensino remoto, horas diárias de trabalho) e roteiro com perguntas semiestruturadas (sobre as formas de lidar com as TICs, facilidades e dificuldades, soluções, atendimento das necessidades, relação com os estudantes, rotinas e cotidiano do trabalho). Exploraram-se quem sabe, de onde sabe, o que sabe, como sabe e o efeito^(10)^.

### Análise dos dados

A análise dos dados do perfil psicosociodemográfico foi feita por meio de frequências absolutas e relativas. As entrevistas transcritas formaram o *corpus* de dados qualitativos com 15 Unidades de Contexto Inicial (UCIs). Este texto único foi processado no *software* Alceste, que aplica testes estatísticos no rastreamento de palavras para a realização de análise lexical contextual de um conjunto de segmentos de texto. Tais testes buscam a frequência e a coocorrência das palavras nos discursos processados, separando os segmentos de textos que fazem sentido nas explicações das ideias veiculadas pelos participantes sobre o objeto/fenômeno investigado. Dessa forma, identificam-se diferentes pontos de vista coletivos que possibilitam captar as representações sociais^(14)^.

Mediante associação estatística das palavras nos segmentos de textos, o *software* gera dendrogramas chamados de Classificação Hierárquica Descendente (CHD), que hierarquiza, a partir de um valor de *Phi*, as palavras mais significativas das classes lexicais e forma listas com as formas reduzidas (radicais) e as formas completas correspondentes. A compreensão das palavras é feita com a leitura dos segmentos de textos as quais elas pertencem, chamados de Unidades de Contexto Elementar (UCEs). O objetivo foi alcançado com a análise de duas classes lexicais. A classe 1 trata dos desafios para adequação a uma nova rotina de vida diante da pandemia, e a classe 2 aborda as interferências vivenciadas pelos docentes idosos na pandemia, sendo essas as categorias de análise.

## RESULTADOS

Majoritariamente, a pesquisa contou com mulheres (13-87%) e apenas dois (13%) homens. A idade variou entre 60 e 78 anos. Três (20%) são docentes do ensino médio; um (7%) é do fundamental; e 11 (73%) são do ensino superior. Nove (60%) possuem pós-graduação *stricto sensu*; nove (60%) possuem atuações exclusivas em instituições públicas; 13 (87%) trabalham mais de oito horas por dia; e 11 (73%) possuem ensino superior. Quanto ao estado conjugal, nove (60%) são casadas; quatro (26%) divorciadas; uma (7%) é viúva; e uma (7%) é solteira. Quanto à família nuclear, novem (60%) residem com o cônjuge; uma (7%) reside com os filhos; três (20%) moram sozinhas; e duas (13%) moram com outras pessoas. Majoritariamente os participantes relataram dificuldades para iniciar as aulas em meio remoto (11-73%).

As classes lexicais 1 e 2 versam como a pandemia de COVID-19 impactou a rotina das pessoas, em especial, dos docentes idosos. Os trabalhadores precisaram se readaptar e reorganizar sua vida pessoal, associando-a à vida profissional, com o trabalho em *home office*. Essas classes retratam a rotina dos professores e como associavam o cuidado com sua saúde e dinâmica adotada no desenvolvimento de aulas remotas. Para compreendê-las, analisou-se a lista de UCEs a ela pertencentes, para apreensão dos contextos em que os léxicos se inseriram, considerando a relação entre as palavras expressas por meio da CHD.

Os desafios para adequação a uma nova rotina de vida foram mais bem evidenciados na classe 1, com 18% (85) do total das UCEs do *corpus* analisado e 119 formas reduzidas de palavras plenas. A análise dos léxicos associados à classe 1 evidenciou que as UCIs tratam da rotina dos docentes durante a pandemia para readequar seu novo modelo de trabalho. Com isso, algumas UCEs apresentam palavras que expressam conforto, agilidade e prazer de estar em casa e trabalhando ao mesmo tempo, enquanto outras referem problemas de saúde devido à quebra de rotina e por passarem muito tempo em uma mesma posição, por exemplo. O léxico de maior associação a esta classe é “hora”, com *Phi* 0,37, como se pode identificar na CHD exposta no Quadro 1.

**Quadro 1:** Palavras representativas da classe 1 conforme o dendrograma da Classificação Hierárquica Descendente gerada pelo Alceste

Classe 1 - 85 Unidades de Contexto Elementar	
**Forma completa e frequência**	**Valor de *Phi***
hora (15) horas (23)	0,37
tempo (43)	0,27
sair (12)	0,27
casa (32)	0,26
época (7)	0,26
horária (1) horário (7) horários (2)	0,25
almoçar (1) almoço (6)	0,24
trabalha (2) trabalhamos (1) trabalhando (5) trabalhar (6) trabalhava (3)	0,24
física (13) físicas (1)	0,23
deslocamento (9)	0,23
acaba (3) acabava (3) acabei (2) acabou (3)	0,23
preparar (8) preparo (2)	0,22
jeito (5)	0,22
noite (5)	0,22
come (3) comer (5)	0,21
rua (6)	0,21
procurar (4) procurei (1) procuro (2)	0,21
acordar (2) acordo (3)	0,19
começa (2) começar (1) começaram (1) começava (4) começavam (2)	0,19
dia (15) dias (1)	0,19
sai (2) saía (3)	0,19
caminhada (3) caminham (1) caminhar (2) caminhava (2)	0,19
atividade (13) atividades (5)	0,19
ir (9)	0,17
chega (1) chegar (3) chegava (4) chegou (1)	0,17
vida (6) vidas (1)	0,17
correr (2) corria (1)	0,17

*Fonte: relatório detalhado por Alceste, 2023.*

A pandemia e o deslocamento da sala de aula institucional para o ambiente residencial implicaram uma importante readequação da rotina dos professores na pandemia, com muitas horas seguidas de trabalho e de tela.

*A gente está em casa, não perde tempo, toda hora vai tomar café, levanta. Normalmente, as reuniões começavam às nove. Eu fiquei muito tempo no computador, fica ruim. Praticamente, o dia inteiro em reunião, foi super muito preenchido.* (UCI 13)

*Literalmente trabalhando. Às vezes, era sete horas da manhã e estava sentada na frente do computador trabalhando, e na hora do almoço, fiz muitas vezes, comendo, fechava a câmera e a gente continuava trabalhando.* (UCI 7)

A readequação da rotina gerou sobrecarga de trabalho e reordenou os hábitos diários de autocuidado, em especial com a alimentação e atividades físicas. Nesse sentido, a saúde física e a saúde mental durante e após a pandemia são aspectos a serem considerados em relação à saúde ocupacional dos docentes, mormente pelo fato de serem pessoas idosas.

[No presencial] *O dia todo sobe e desce escada; vai pra um lugar; vai pro outro; come nas horas que tem que comer. No ensino remoto, toda hora você vai à cozinha, come uma coisa fora do tempo, não faz atividade física direito, não tem uma rotina. Não fiz absolutamente nada de atividade física. Saía da cadeira para comer e beber água, um horror. Assim que permitiu, eu voltei a fazer atividades.* (UCI 14)

*Eu parei de fazer caminhada. Eu tinha dores de coluna, tive comprometimento de panturrilha, encurtamento, fiquei quase sem andar porque ficava sentada, não conseguia andar.* (UCI 7)

*Às vezes, dez horas da noite e eu trabalhando, marcando reuniões de orientação dos alunos oito, nove horas da noite, porque eles tinham outras atividades. Isso fez com que houvesse um desgaste físico e também um pouco de desgaste mental.* (UCI 10)

Entre os desafios com a tecnologia, os docentes idosos precisaram lidar com novos planejamentos diante do reordenamento de horários para fazer frente à nova realidade laboral que estavam vivenciando.

*O planejamento de horário não é o mesmo quando em sala de aula. Como eu me empolgo, começo a fazer e esqueço de comer. Eu esquecia de tudo. Eu sento ali e vou, porque, se eu parar, parece que vou perder o foco e a motivação, porque ainda dá para aprender bastante coisa. Está facilitando o trabalho. Ainda bem que a gente estava na pandemia, tinha o dia inteiro em casa, porque às vezes vou preparar uma aula, levava um dia inteiro, virava parte da noite pesquisando, porque eu só sei trabalhar com livros.* (UCI 6)

Observa-se nos resultados uma dialética na nova modalidade de ministrar aulas, com aspectos positivos e negativos. Por um lado, trabalhar por meio remoto faz com que os professores economizem tempo no deslocamento, mas, por outro, o confinamento não era um estado desejado.

*Às vezes, ficava na cama e pensava quando é que a gente vai voltar para aquele mundo de engarrafamento de levar duas horas até o trabalho. Será que a gente acostuma com isso de novo? Mas, ao mesmo tempo, a gente não estava querendo ficar daquele jeito.* (UCI 13)

A economia de tempo se apresenta com duas faces, pois o que sobra do deslocamento não ocorrido reverte em mais horas de trabalho.

*Porque você gastava um tempo muito maior em função do trabalho; tinha que se preparar para chegar e sair do trabalho e chegar em casa. Eu não ocupo tempo para me arrumar, me maquiar, procurar uma roupa, um sapato, sair e pegar metrô, então esse tempo que eu usava para isso, nós usamos pra quê? Literalmente trabalhando.* (UCI 7)

Os docentes também indicaram que, para os alunos, o ensino remoto trouxe algumas vantagens, como a possibilidade de estudar em horários e locais que não são os normativos do ensino presencial, além de possibilitar economia financeira no deslocamento, aumentando a possibilidade de que mais alunos pudessem ser inseridos nas instituições de ensino.

*Custo altíssimo para ir e vir da faculdade. O ensino remoto pode ser bom porque pode inserir muito mais alunos na universidade. O mal é não ter uma rotina adequada, come demais, engorda demais, muda seus hábitos, porque, no dia a dia, é correria pra acordar, se arrumar, tomar o café, às vezes até no carro e ir pro trabalho.* (UCI 14)

No entanto, consideram que os alunos precisam se readaptar ao ensino presencial pós-pandemia, com a disciplina que o espaço físico da sala de aula exige.

*Eles* [os alunos] *estão reaprendendo a viver em sociedade; não conseguem ficar tantas horas sentados no mesmo lugar, porque, em casa, estavam no sofá, cama; sentavam de qualquer jeito, agora tem que passar cinco horas sentado, tudo está mais difícil.* (UCI 6)

As interferências vivenciadas pelos docentes na pandemia foram mais bem evidenciadas na classe 2, que tem 42% (192) do total das UCEs, constituindo o *corpus* de análise, tornando-se a classe mais densa. É constituída de 135 formas reduzidas de palavras plenas encontradas. A análise dos léxicos associados à classe 2 evidenciou os mecanismos utilizados tanto pelos professores quanto pelos alunos, para terem comunicação e troca de informações diante do novo modelo de aula, então são vistas palavras como microfone, chamadas *online*, janelas abertas e internet lenta, pois foram fatores que interferiram diretamente no cotidiano. O léxico de maior associação a esta classe é aula, como se pode identificar no Quadro 2.

**Quadro 2:** Palavras representativas da classe 2 conforme o dendrograma da Classificação Hierárquica Descendente gerada pelo Alceste

Classe 1 - 192 Unidades de Contexto Elementar	
**Forma completa e frequência**	**Valor de *Phi***
aula (94) aulas (47)	0,32
pergunta (5) perguntando (1) perguntar (2) perguntaram (1) perguntas (5)	0,24
alunos (59)	0,22
plataforma (16) plataformas (6)	0,23
*online* (37)	0,21
manda (5) mandar (2) mandaram (1) mandava (2) mandavam (1) mando (2)	0,18
usava (18) usávamos (1)	0,16
curso (14) cursos (2)	0,16
iniciar (2) iniciaram (2) início (13) iniciou (2)	0,15
fazendo (17)	0,15
suporte (7)	0,14
interage (3) interagia (1) interagiam (2) interagir (1)	0,14
fala (3) falam (2) falando (7) falar (6) falava (10) falavam (3) falei (4) falo (2) falou (2)	0,13
aluna (1) alunas (1) aluno (35)	0,13
entra (2) entrada (2) entramos (2) entrar (7) entrava (4) entravam (2) entro (3)	0,13
cama (1) câmera (17)	0,12
rio (6)	0,13
sala (25) salas (4)	0,12
jitsi (7)	0,13
virtuais (2) virtual (13)	0,12
confusão (4) confuso (3)	0,13
*notebook* (10)	0,13
compartilha (1) compartilhada (1) compartilhando (1) compartilhar (6)	0,13
aprendizagem (9)	0,13

*Fonte: relatório detalhado por Alceste, 2023.*

Alguns dos desafios relatados pelos professores para ministrar aulas remotas dizem respeito à vida privada do estudante, que veio à tona na sala de aula remota, pois a inabilidade com os microfones trazia para a aula parte do que ocorria nas suas casas. Outros comunicam dificuldades e inabilidades de todos, estudantes e professores, com as ferramentas da plataforma, necessitando de aprendizagem para um melhor manuseio e aproveitamento das aulas.

*Esqueciam o microfone aberto; era criança chorando, cachorro latindo, isso fazia parte das intercorrências, o microfone aberto. Fora isso, os alunos tiveram excelentes rendimentos, porque prestavam atenção. Se o microfone estivesse aberto, eu pedia para fechá-lo; eles obedeciam e prestavam atenção e interagiam na sala de aula.* (UCI 11)

*No início, foi tudo muito confuso. Os alunos não achavam as salas de aula corretas, não conseguiam conexão, atrasavam as aulas, uma experiência não muito boa. Tiveram cursos online e enviavam materiais pra nos ajudar, deram o suporte pra gente.* (UCI 14)

*Os avisos ficavam lá, mas não era eu que colocava, era a coordenadora da disciplina, porque eu só sabia entrar e olhar. Na plataforma Jitsi, eu aprendi rápido, entrar e compartilhar a tela, colocar na tela cheia. No início, eu tinha dificuldade.* (UCI 8)

*Às vezes, a imagem fica ruim. Pra mim, foi um susto, mas hoje olho com bons olhos. Eu me reinventei. E as aulas online, como eu disse, preciso ficar atenta, porque se tiver algum aluno fazendo exercício errado, eu preciso chamar atenção.* (UCI 3)

*Verdadeiramente, assim, as aulas, tinha uma pessoa do departamento de informática da escola que estava nas primeiras aulas, se a gente tivesse alguma dificuldade para mexer no equipamento, dificuldade, eles davam aquele suporte até a gente aprender.* (UCI 9)

Diante das adversidades, reinventar-se foi a estratégia aplicada para cumprir com as atividades laborais durante a pandemia. Esta “reinvenção” foi feita com a ajuda de colegas e com o aprendizado concomitante ao trabalho.

*Quem era mais descolado, falava, sabe aonde eu entro, como vou compartilhar, como ligo isso. Foi no desenvolvimento e até que a gente pegou, mas o ambiente virtual de aprendizagem é bom, mas é meio complicado, eu não dava aula neste ambiente*. (UCI 13)

*Teve pouco apoio psicológico da instituição. Às vezes, faz falta. Tem sempre alguém na escola para dar uma ajuda, um apoio. A gente aqui tem um psicólogo, aí a gente corre pra ele, manda o aluno que está dando problemas, de conversar com o aluno*. (UCI 6)

*Tínhamos grupos pelo WhatsApp. Um ajudava o outro, tentávamos fazer as aulas em dupla e uma ficava interagindo com a turma no chat e outra dando a aula.* (UCI 14)

As preocupações com o aproveitamento das aulas e das provas por parte dos alunos e a detecção de que estar com o *login* ativo e estar *online* não significavam estar presente nas aulas.

*Alguns alunos pareciam online mesmo, e havia aulas que a coordenadora não aceitava que fosse só a carinha. A gente tem que estar online, mostrando o movimento de cada um de vocês* [referindo-se aos estudantes de pós-graduação]; *exigia que fosse assim. No ensino remoto, ela observou que o aluno não estava presente, só tem o rosto.* (UCI 9)

## DISCUSSÃO

Para controlar a pandemia de COVID-19, foram adotadas medidas de distanciamento social, com circulação restrita das pessoas e implantação do trabalho remoto^(15)^. O que se viu foi uma readaptação da rotina de trabalho, tanto para os profissionais, que permaneceram de forma presencial, quanto para os que iniciaram com o teletrabalho^(16)^.

Aos que seguiram na forma presencial, havia o medo de contágio, porém também tinham receio do desemprego e tentaram seguir com a sua rotina, considerada “normal”. Quem trabalhava remotamente teve dificuldade de adaptação, com mudanças na organização e rotina do lar, entendido como local de restauração e descanso, que passou a ser seu novo espaço de trabalho, além das dificuldades em conciliá-lo com as demandas domésticas^(16)^. Serviços administrativos e escolares foram os que mais aderiram a essa modalidade de trabalho^(15)^. O encerramento das atividades envolveu fortemente as instituições educativas. Com isto, exigiu-se que todos os níveis de organização de ensino trabalhassem de forma remota emergencial^(17)^.

Desde o início da pandemia, foi preciso que as pessoas readequassem suas vidas e vivenciassem mudanças bruscas e significativas no cotidiano. O tempo gasto para se preparar e se deslocar para os espaços de trabalho presencial foi investido no próprio trabalho, o que alterou a rotina dos professores idosos. Em relação a comportamentos de saúde, observa-se que o cuidado com o estilo de vida é uma recomendação para manter e promover a saúde de pessoas idosas, sendo os exercícios físicos, a alimentação, o descanso e o lazer importantes pilares a serem resguardados^(18)^. Justamente tais cuidados foram comprometidos com a implantação do trabalho remoto, sendo uma das faces negativas dessa fase, à luz das representações dos professores idosos.

Pesquisa realizada sobre comportamento sedentário na pandemia de COVID-19, com adultos e pessoas idosas de Campo Grande, no Mato Grosso do Sul, teve participação de 1.907 pessoas e evidenciou o excesso de uso de telas por 86,7% dos participantes em geral, sendo que as pessoas idosas apresentaram menor frequência de tempo de tela, mas maior proporção de tempo sentado^(19)^. Esta pesquisa não foi feita com docentes em ensino remoto, não tendo a sua citação a pretensão de estabelecer comparações com os resultados da pesquisa objeto deste artigo. Não obstante, aponta para a tendência de sedentarismo de pessoas idosas na pandemia e permanência de logo tempo sentado, comportamento próprio de quem trabalha por muitas horas em computadores, o que indica baixa carga de atividades físicas nesse período de reclusão em domicílio.

Pesquisa realizada com 1.115 professores holandeses de todos os níveis de ensino, cuja maioria era do sexo feminino (76,7%) e idade entre 19 e 69 anos (média de 43 anos), indicou comprometimento da vida pessoal com o trabalho remoto, destacando a necessidade de equilíbrio entre o mundo do trabalho e o privado em relação às horas trabalhadas, melhores instalações para o trabalho em casa e à conciliação com as demais responsabilidades e o autocuidado^(20)^. No Brasil, pesquisa com 17 docentes de uma universidade pública mostrou que as modificações na rotina do trabalho implicaram a dificuldade de limitar as horas de trabalho e de descanso^(21)^.

O impacto na saúde mental e a necessidade de apoio psicológico emergiram no conjunto das preocupações dos docentes idosos. Epidemias geram grande impacto na saúde mental e no caso da COVID-19, e a falta de pausas e descansos foi apontada como um dos fatores para este comprometimento^(22)^. A rotina equilibrada entre a atenção a si, ao trabalho e à família, e a manutenção de atividades físicas, desde que resguardados os cuidados preventivos à infecção, são medidas recomendadas para manter a boa saúde mental^(22)^. Os docentes idosos em ensino remoto ressaltaram que a rotina diária, o excesso de trabalho e o sedentarismo comprometeram o seu cotidiano. Pesquisa realizada sobre as representações sociais do confinamento junto a profissionais e estudantes captados por meio de redes sociais evidenciou que os efeitos do confinamento se fizeram sentir na saúde mental de todos, mas especialmente no grupo de estudantes^(23)^, o que indica ser este um tema candente para estudos na interseção dos campos da educação e da saúde.

Além das adaptações no dia a dia, reorganizações de ordem estrutural relativas à reconfiguração do trabalho docente demandaram aprendizagens de novas habilidades para lidarem com as tecnologias que o trabalho remoto exige^(24)^. A readequação e a reinvenção didática para ministrar as aulas implicaram mudanças no cotidiano doméstico, pois as aulas virtuais alteraram a rotina pessoal. Muitos informaram a necessidade de aprender a manusear e acessar as plataformas tecnológicas, e se depararam com problemas de conexão, alunos sem possibilidade de acesso, dificuldades de se comunicar devido a ruídos externos, entre outros.

Em consonância com os problemas que os docentes idosos vivenciaram, os professores russos que atuaram no ensino remoto também indicaram que a falta de preparo para lidar com as tecnologias digitais, de recursos e de tempo para se preparar foi o principal desafio a enfrentar no ensino remoto emergencial exigido na recente pandemia^(20)^. Professores e estudantes tiveram dificuldades, e isso implicou interferências de diversas ordens, principalmente por conta dos microfones abertos e dos atrasos no início das atividades. O trabalho docente por si gera muitas demandas^(24)^, e quando o docente se vê em uma situação em que precisa reaprender o seu trabalho perante tecnologias que não domina após anos de trabalho, e mesmo num contexto adverso como o de uma pandemia, as emoções vêm à tona. Primeiro vem o susto, e depois, a aceitação e a reinvenção.

Representações sociais são quase tangíveis, circulam nas falas, gestos, encontros e se impregnam no universo cotidiano, correspondendo à substância simbólica da sua elaboração e à prática que produz tal substância^(11)^. São saberes práticos que se organizam e se constroem diante das novidades do cotidiano, mobilizando afetos e influenciando comportamentos. Tal construção ocorre pelos processos de ancoragem, que assimila a novidade no sistema de pensamento já existente, e pela objetivação, que, ao acolher tal novidade, a integra nas ações cotidianas^(10)^.

Os docentes se depararam com a novidade de um novo modo de trabalhar, em novos espaços e com ferramentas que eles já conheciam - os computadores -, mas não como sala de aula. Habituados com salas de aula física tiveram que se transportar para o meio virtual e lidar com esta nova situação, que se apresentou como uma novidade que demandou novas formas de atuação e de como se comportar neste novo espaço, com a transformação de seus saberes sobre como ministrar aulas em um espaço não físico para estudantes que nem sempre estão visíveis aos seus olhos.

O deslocamento da sala física para a virtual exigiu atenção diferenciada, com preocupações que antes os docentes não tinham: acostumarem-se com a participação dos alunos somente por meio da voz, já que muitos não abriam as câmeras, o que requeria um novo aprendizado sobre a presença a partir do sentido da audição e não da visão; verificar os problemas de conectividade; e adequar as propostas das aulas aos recursos disponíveis na plataforma utilizada pela instituição.

O trabalho docente não se limita à sala de aula, e vale destacar que, além das novas demandas, foram acrescentadas aquelas já existentes no âmbito do trabalho docente, principalmente quando atuamos no ensino superior, que incluem a liderança de grupos de pesquisa, orientações de trabalhos acadêmicos, coordenação de projetos de extensão, entre outras^(25)^.

A questão de o ensino remoto possibilitar maior inclusão de estudantes foi destacada no contexto da economia de gastos com os deslocamentos. No entanto, o fechamento das escolas acarretou altos custos sociais e econômicos para a população em diferentes esferas^(26)^. O impacto foi particularmente grave para os mais vulneráveis e marginalizados e suas famílias, com a interrupção da aprendizagem e consequente perda de oportunidade de crescimento e desenvolvimento. As desvantagens são desproporcionais para os estudantes menos privilegiados, pois tendem a ter menos oportunidades educacionais além da escola.

No Brasil, grande parte das escolas públicas esteve fechada durante quase todo o ano letivo em 2020, e reabriram somente em 2021, ao contrário das escolas privadas, que reabriram em setembro e outubro de 2020, o que mostra as desigualdades de acesso para os alunos^(27)^. Estudantes de escolas públicas, do ensino fundamental ao superior, sentiram muito mais o impacto da mudança repentina das aulas, pela falta de equipamentos tecnológicos e acesso à internet em sua residência, além de dificuldades para manusear os aplicativos. Um aspecto preocupante é o maior impacto da pandemia nos estudantes considerados vulneráveis e marginalizados, que podem ficar mais atrasados^(28)^. Um dos temas que a sociologia da educação discute é a relação entre o nível socioeconômico dos alunos e o sucesso escolar, pois o núcleo social tem um significado importante no desempenho dos alunos.

Os resultados apresentados se coadunam com os de uma pesquisa realizada com docentes de instituição de ensino superior da região Sul do Brasil que, apesar de não ser voltada para docentes idosos, indicou desafios semelhantes aos dos idosos sobre o processo de ensino aprendizagem, a demanda por aprender novas tecnologias digitais e os impactos na saúde mental dos docentes^(29)^.

Vencer o medo e a insegurança para aprender a lidar com as tecnologias permeou as experiências dos docentes no ensino remoto, e a interferência na vida privada, o trabalho e o lazer marcaram as experiências dos docentes do sul, como também dos docentes idosos participantes desta pesquisa. As dificuldades com o manejo de equipamentos e plataformas digitais foram sentidas por diferentes docentes, e o medo, angústia e incertezas sobre como seria o trabalho docente por meio remoto os acompanharam nesta jornada de familiarização e readequação do trabalho^(30)^. Sobre o fenômeno do medo, destaca-se que o medo do desconhecido é um tipo específico que se apodera das pessoas em tempos de crise^(31)^. Como estado afetivo, o medo estrutura a resposta para que a pessoa se adapte às situações, o que, sendo racional, permite reações diante de situações que se apresentam como novas^(31)^.

As estratégias acionadas para o desenvolvimento de um trabalho de qualidade que cumprisse com o propósito do ensino-aprendizagem também precisaram ser reformuladas, já que o espaço do ensino mudou. A verificação da efetiva presença do estudante no meio virtual e o fortalecimento da solidariedade entre os professores foram destacados. As dúvidas e incertezas em relação ao contexto e à nova modalidade de ensino mobilizaram os afetos/emoções na produção de sentidos sobre o trabalho remoto, com acionamento de conhecimentos prévios, normas, valores, ideologias e sistemas de crenças que se compartilham socialmente. Vê-se o papel que a emoção ocupa no trabalho cognitivo na mobilização de saberes sociais para reinterpretar o mundo e nas transações (acordos, práticas) com o meio^(31)^. A qualidade do ensino remoto figura nas percepções de docentes^(21,32)^. Professoras e coordenadoras pedagógicas do curso de mestrado de uma universidade da cidade de São Paulo manifestaram preocupações com a aprendizagem de educandos pelos diferentes perfis de desigualdade de idade, currículo, estratégias de ensino e acesso à internet^(32)^.

O senso de empatia e a solidariedade para com outros docentes se fizeram sentir neste enfrentamento. A gestão escolar e a relação com os colegas de trabalho emergiram das necessidades dos professores holandeses em trabalho remoto na pandemia, com mais orientações por parte das instituições em relação ao próprio processo de trabalho nesta nova modalidade e à preocupação com colegas que precisam de ajuda e apoio, em um trabalho mais colaborativo^(20)^.

As dificuldades com as tecnologias e a sua representação como desafio permeiam o conjunto de resultados desta e de outras pesquisas realizadas na pandemia sobre o tema. A pandemia trouxe à tona a tensa relação entre o trabalho de professores idosos e as tecnologias de ensino remoto. Para corroborar esta afirmação, pesquisa realizada nas Filipinas sobre notícias veiculadas em vídeos envolvendo professores idosos e aprendizagem *online* de emergência implantada na pandemia de COVID-19 mostrou que os professores se empenharam para se adaptar a este tipo de ensino, empenhando-se na aprendizagem do manuseio das ferramentas e no atendimento das expectativas. Os autores indicam que, nas Filipinas, os professores idosos representam um dos desafios a serem enfrentados pela administração escolar e pelo governo. Esses professores precisam de mais atenção para adquirir e melhorar habilidades técnicas e confiança para trabalhar no ensino remoto^(8)^.

A complexidade de uma das plataformas usadas pela instituição foi traduzida como um ambiente complicado que culminou na opção de não uso. Esta situação também está pontuada na pesquisa realizada nas Filipinas, pois uma das recomendações foi a produção de plataformas de manuseio simples, para colaborar com professores idosos nessa modalidade de ensino^(8)^.

Pesquisa em teses e dissertações produzidas entre 2017-2021 sobre formação continuada de professores e tecnologias digitais analisou 44 trabalhos, sendo a metade produzida na região Sudeste, centradas em práticas pedagógicas em sala de aula, que mostra a educação básica na centralidade dos estudos^(33)^. Logo, a educação permanente é uma boa estratégia para preparar professores ao uso de recursos digitais, não somente para o ensino remoto, que na pandemia teve caráter emergencial, mas porque o campo digital na educação é um caminho que só tende a crescer.

### Limitações do estudo

Esta pesquisa foi realizada com docentes de um munícipio da região Sudeste. O Brasil é um país continental, e um debate mais ampliado com experiência de docentes idosos de outras regiões permitiria considerar realidades socioculturais diversas e proposições mais abrangentes para a área.

### Contribuições para as áreas da enfermagem, saúde ou políticas públicas

O envelhecimento populacional é uma realidade contemporânea, assim como os avanços tecnológicos, apesar dos recursos de informação e comunicação. A educação é uma área de importante aplicação de tecnologias para diversificar estratégias de ensino-aprendizagem, seja para o ensino remoto ou a distância, e para difundir informações e popularização da ciência. Incluir os docentes idosos nesse debate e prepará-los para essas práticas é oportuno e necessário, haja vista o contingente de pessoas com 60 anos ou mais que se apresenta no mercado de trabalho, e esse reconhecimento é fundamental para a qualificação e a saúde deste grupo populacional.

## CONSIDERAÇÕES FINAIS

A readequação da rotina implicou mais horas de trabalho, pois a economia de tempo de deslocamento resultou em sobrecarga laboral. A perda da rotina resultou em mais consumo de alimentos, aumento de peso, sedentarismo e comprometimento da saúde mental. Os desafios do novo espaço de sala da aula foram enfrentados com a aprendizagem das funcionalidades da plataforma, traduzido como um processo de se “reinventar” enquanto professor no desenvolvimento do trabalho. Houve pouco apoio psicológico por parte das instituições. A inclusão de estudantes, pela economia dos gastos com deslocamento, e o trabalho colaborativo foram aspectos positivos. Evidencia-se a necessidade de investimentos em atualizações do corpo docente e maior assistência aos docentes idosos nas suas dificuldades para vencer as limitações.

## Data Availability

Não se aplica.
